# Incidence of Post-Operative Complications and Factors Influencing Their Occurrence in Patients with Sickle Cell Disease in a Low-Income Country: A Case Study of Cameroon

**DOI:** 10.3390/jcm11030780

**Published:** 2022-01-31

**Authors:** Dominique Djomo Tamchom, Charlotte Eposse Ekoube, Basile Essola, Serge Nga Nomo, Fleur Samantha Benghiat, Luc Van Obbergh

**Affiliations:** 1 Faculty of Health Sciences, University of Buea, Buea 63, Cameroon; 2 Faculty of Medicine, Free University of Brussels, 1070 Brussels, Belgium; basile.essola@ulb.ac.be (B.E.); samantha.benghiat@erasme.ulb.ac.be (F.S.B.); luc.van.obbergh@erasme.ulb.ac.be (L.V.O.); 3 Department of Anaesthesiology and Intensive Care, Douala Gynaeco-Obstetric and Paediatric Hospital, Douala 7072, Cameroon; 4 Department of Anaesthesiology, Erasme Hospital, 1070 Brussels, Belgium; 5 Faculty of Medicine and Pharmaceutical Sciences, University of Douala, Douala 2701, Cameroon; eekoubec@yahoo.fr; 6 Sickle Cell Treatment Centre, Laquintinie Hospital, Douala 4035, Cameroon; 7 Higher Institute of Medical Technology, Yaoundé 188, Cameroon; sergesvivier@yahoo.fr; 8 Department of Anaesthesiology and Intensive Care, Essos Hospital Centre, Yaoundé 441, Cameroon; 9 Department of Haematology, Erasme Hospital, 1070 Brussels, Belgium

**Keywords:** patient with sickle cell disease, anaesthesia, surgery, post-operative complications, incidence

## Abstract

This study aimed to analyse post-operative complications and possible factors influencing their occurrence in the management of patients with sickle cell disease in a low-income country. We prospectively collected data regarding the management of patients with sickle cell disease requiring anesthesia for surgery in 11 Cameroonian hospitals from 1 May 2019 to 30 April 2021. The data were analysed using descriptive statistics and a binary logistic regression was used to determine the dependence between the variables. A total of 124 patients with sickle cell disease were enrolled; 64 were male and 60 female, giving a sex ratio of 0.93. The rate of post-operative complications was 23.4% (29/124) and the death rate was 3.2% (4/124). The female subjects had more complications than the male subjects *p* < 0.05. The number of vaso-occlusive crises experienced per year showed a significant impact on the occurrence of post-operative complications *p* < 0.05. Laparoscopic surgery had fewer post-operative complications 5/46 (10.9%) than laparotomy 14/43 (32.5%). The surgical technique for the abdominal procedures had a significant impact on the occurrence of post-operative complications *p* < 0.05. The type of surgery (*p* = 0.198) and the anaesthesia technique (*p* = 0.225) did not show a significant impact on the occurrence of post-operative complications. Particular attention should be paid to female patients with sickle cell disease as they are more likely to experience post-operative complications, as well as to the frequency of vaso-occlusive crises, which are also predictive of post-operative complications. Opting for laparoscopic surgery whenever possible would help to reduce post-operative complications.

## 1. Introduction

Sickle cell (SC) disease is the most common genetic pathology in the world [1]. About 200,000 people are born with this disease each year and the regions with the highest rates are Africa, the Mediterranean, and Asia, where its prevalence is estimated at 2 to 6% of the population [2]. In Cameroon, the prevalence of sickle cell traits is 22.3% and the prevalence of SS homozygous varies from 1.7% to 9%, depending on the region [3]. Despite the complex pathophysiology and the diverse clinical picture, considerable progress has been made in the understanding and management of sickle cell disease [4]. Thanks to improved neonatal screening and treatment, many patients with sickle cell disease survive to adulthood and present themselves more frequently for surgery [2]. It is in this situation that the anaesthesiologist is concerned, especially since 15 to 30% of patients undergoing surgery suffer from post-operative complications [5,6,7]. Surgery exposes the sickle cell disease patient to an increased risk of disease-related complications [8], requiring meticulous perioperative clinical care, including adjusted anaesthesia management, which has been widely described. Most of the data are issued from western countries and only a few from the sub-Saharan area, where facilities are limited, even though they represent most of the SC patients undergoing surgery, suggesting that the data issued from the former countries could be biased [7,9,10]. Therefore, this study is crucial as it contributes to the body of research by analysing post-operative complications and possible factors influencing their occurrence in the perioperative management of sickle cell patients in a low-income country.

## 2. Methods

After obtaining the ethical clearances from our Institutional Ethics Committee and the National Ethics Committee for Research for Human Health (CNERSH), we prospectively collected data regarding the management of sickle cell patients requiring anesthesia for surgery in 11 Cameroonian hospitals from 1 May 2019 to 30 April 2021. The pre-determined data collection sheet was handled by anaesthesiologists working in hospitals (11) classified as levels 1 and 2 (the highest levels of the health pyramid in Cameroon), from t pre-anaesthetic consultation to post-anaesthetic follow-up. Only facilities with at least one fully qualified anaesthesiologist were included, given the limited number of anaesthesiologists practicing throughout the territory, which was about 50 physicians per 25 million inhabitants in 2019. The classification of risk (minor, intermediate, and high) according to the type of surgery was performed and associated with each collection sheet.

All patients with major sickle cell syndrome (referring to more or less severe clinical manifestations, characterised by SS homozygosity and SC composite heterozygosity) admitted to one of the targeted health facilities, who had been under the care of an anaesthesiologist and had undergone surgery, were included in this study. The major sickle cell syndrome combines three main categories of clinical manifestations: chronic haemolytic anaemia; extreme susceptibility to infections; and vaso-occlusive phenomena. It differs from asymptomatic (AS) forms. Those who did not undergo surgery and those who withdrew their consent were excluded.

**Figure 1:**
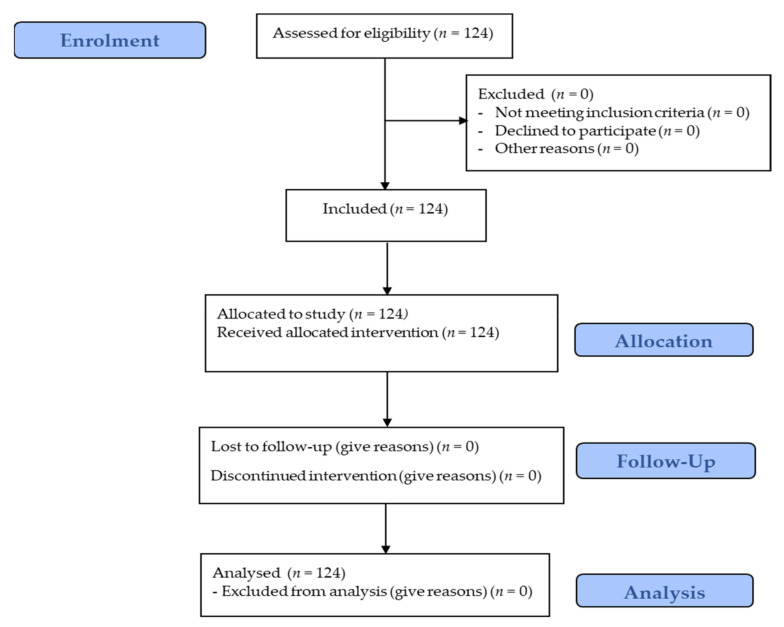
CONSORT 2010 Flow Diagram.

The characteristics of the patients with sickle cell disease were determined using the patients’ medical records or by the patients’ reports when records were not available. The post-operative complications that occurred were recorded as well. Post-operative complications were those that occurred between the end of surgery and post-operative discharge or those that required patients to return to the hospital within 15 days of discharge. Mortality was defined perioperatively. We then looked at the factors that could influence the occurrence of these complications in this study population. As already described, the data were collected on a previously determined sheet, and as such, the main variables were: regular treatment with Hydroxyurea, number of vaso-occlusive crises experienced per year, level of risk associated with surgery, pre-operative transfusion, length of hospital stay before surgery, type of surgery, duration of surgery, surgical technique, ASA classification, context of surgery, and type of anaesthesia. We used convenience sampling to obtain a size sample (Figure 1).

The statistical analysis was performed using IBM SPSS version 23 software (International Business Machines Corporation, New York state, USA). The results were expressed as a percentage number and as an average standard deviation and interval. Binary logistic regression was used to determine the dependence between the variables. The rules of thumb of the binary logistic regression for this study stats that significant variables have a significant impact on the occurrence of post-operative complications in a patient with sickle cell and variables that are not significant do not have a significant impact on the occurrence of post-operative complications. A value of *p* < 0.05 represents a statistically significant dependence at the 5% level.

## 3. Results

A total of 124 patients with sickle cell disease were enrolled; 64 were male and 60 female, giving a sex ratio of 0.93. The rate of post-operative complications was 23.4% (29/124) and the death rate was 3.2% (4/124). The mean age of the population was 20.5 ± 7.1 years, with a minimum and maximum of 5 and 47 years, respectively. The mean number of vaso-occlusive crises experienced per year was 2.1 ± 1.1, with a minimum and maximum vaso-occlusive crisis of 1 and 5, respectively. The mean length of pre-operative hospital stay was 2.1 ± 1.4 days, with a minimum and maximum of 1–14 days, respectively. The other characteristics of the study population are listed in Table 1.

Considering the type of surgery in our overall study population, the rate of post-operative complications was higher after orthopaedic surgery 9/30 (30%). Those who underwent laparoscopic surgery for abdominal procedures had fewer post-operative complications 5/46 (10.9%) than those who underwent laparotomy 14/43 (32.5%). Patients with sickle cell disease who received locoregional anaesthesia had more post-operative complications 12/41 (29.3%) than those who received general anaesthesia 17/83 (20.5%).

Female subjects had more complications than male subjects *p* < 0.05.

The number of vaso-occlusive crises experienced per year showed a significant impact on the occurrence of post-operative complications *p* < 0.05.

The length of pre-operative hospital stay (*p* = 1) and pre-operative transfusion (*p* = 0.989) had no impact on the occurrence of post-operative complications.

Emergency surgery (*p* = 0.721) and anaesthesia technique (*p* = 0.225) did not have a significant impact on the occurrence of post-operative complications.

The type of surgery (*p* = 0.198) and the duration of surgery (*p* = 0.194) did not show a significant impact on the occurrence of post-operative complications.

The surgical technique used for the abdominal procedures had a significant impact on the occurrence of post-operative complications (*p* < 0.05).

The data from the logistic regression are presented in Table 2.

## 4. Discussion

To the best of our knowledge, very few, if any, recent studies have addressed the issue of post-operative complications in patients with sickle cell disease in a diverse population of children and adults simultaneously and for a variety of surgical indications. This study showed that the post-operative complications and their incidences observed in carriers of major sickle cell syndrome in a resource-limited setting were no different from those seen elsewhere. Length of pre-operative hospital stay, pre-operative blood transfusion, emergency context, type of surgery, and type of anaesthesia did not have a significant impact on the occurrence of these complications. However, gender, number of vaso-occlusive crises experienced per year, and surgical technique used influenced the occurrence of post-operative complications.

## 5. Incidence of Post-Operative Complications in Sickle Cell Patients

The post-operative period is particularly critical, with the highest incidence of complications [11]. The main complications (febrile episodes, haemolysis, vaso-occlusive crisis, thromboembolic accidents, and acute chest syndrome) are characterised by hypoxia, hypothermia, and pain [12,13]. Their incidence varies according to the type of procedure and the medical-surgical team. The treatment is based on adequate hydration, oxygen therapy, broad-purpose antibiotic therapy, or even erythrocyte transfusion. All these complications were observed in our series, independently of the type of surgery performed. The overall incidence of post-operative complications of 23.4% found here is close to that reported by some authors, although most of these authors dealt with only one type of surgery.

In the series of laparoscopic cholecystectomy in sickle-cell-disease adults performed between 1996 and 2006 in Saudi Arabia [14], 31/427 (7.3%) of patients had complications, including 19/31 (4.5%) vaso-occlusive seizures; 8/31 (1.9%) acute chest syndromes; 4/31 (0.9%) cases of superficial infection of the surgical wound; and no deaths. In another series of 42 laparoscopic cholecystectomies performed in Senegal [7] between 1998 and 2002, the overall rate of complications noted was 16.7%, with 3 cases (7.1%) of vaso-occlusive crises; 2 cases (4.7%) of acute chest syndrome; 2 (4.7%) cases of post-operative infection; and no deaths. In 138 orthopaedic procedures performed on 118 patients with sickle cell disease in a multicentre study [15], there was an overall rate of 67% of serious complications, and sickle cell events (acute chest syndrome or vaso-occlusive crisis) occurred in 17% of cases. Two patients (1.7%) died following surgery. In a series of cholecystectomies in patients with sickle cell disease in Jamaica [16], the mortality rate was 7.4%.

The results observed in these studies are comparable to ours, in which the complications recorded and their proportions are practically the same. However, it should be noted that there were no deaths in the Saudi Arabian and Senegalese cholecystectomy series. In the orthopaedic surgery multicentre series, the death rate was less than half of ours and in the Jamaican cholecystectomy series, the death rate was double ours. As already mentioned, the previous studies focused only on one type of surgery in patients with sickle cell disease. We included different indications for surgery to analyse the influence of this factor on the incidence of post-operative complications in patients with sickle cell disease.

## 6. Elements That Did Not Have a Significant Impact on the Occurrence of Post-Operative Complications

The pre-operative clinical condition of patients with sickle cell disease may require extensive preparation in advance, since these patients are admitted to hospital for elective surgery several days or weeks before the procedure. However, in our study, the length of pre-operative hospital stay was not a predictive factor for the occurrence of post-operative complications, where the maximum observed pre-operative length of stay was 2 weeks. This is in line with the results reported by some authors. A review of a series of patients with sickle cell disease who underwent cholecystectomy between 1978 and 1991 to assess their perioperative management and clinical outcome [17] showed that these patients were pre-operatively prepared over 2 to 8 weeks, and there were no apparent sickle cell-disease-related vaso-occlusive events or late complications.

Although there is currently no consensus on the benefit of pre-operative transfusion for patients with major sickle cell syndrome [18], their anaesthesia preparation frequently includes the transfusion of red blood cells to reduce the risk of morbidity and mortality associated with the procedure [19]. The purpose of blood transfusion in sickle cell disease is to rapidly decrease the proportion of red blood cells containing haemoglobin S, and thus to prevent harmful pathophysiological cascades. In a study conducted in a reference centre for genetic red blood cell diseases [18], on patients with major sickle cell disease syndrome who underwent cholecystectomy between January 2009 and March 2012, there was no difference in complication rates between the transfused and non-transfused groups. In a cohort of paediatric patients with sickle cell disease undergoing abdominal surgery [20], there was no clear association between post-operative complications and transfusion approach or pre-operative haematocrit level. These results are similar to those in our series. Controversy continues regarding whether and when (pre-operatively or post-operatively) patients with sickle cell disease should receive transfusions and which anaesthetic technique (regional or general) confers benefits [2].

The anaesthetic management of patients with sickle cell disease is well defined [4,21]. Compliance with safety rules is essential as in any form of anaesthesia, general or loco-regional. No specific drug protocol is recommended. Our study showed more frequent use of general anesthesia in these patients. This could be due to the choices and habits of anaesthesiologists, but also to the predominant type of surgery, which was abdominal and, most frequently laparoscopic. However, 13% of the surgeries performed were caesarean sections.

**Figure 2:**
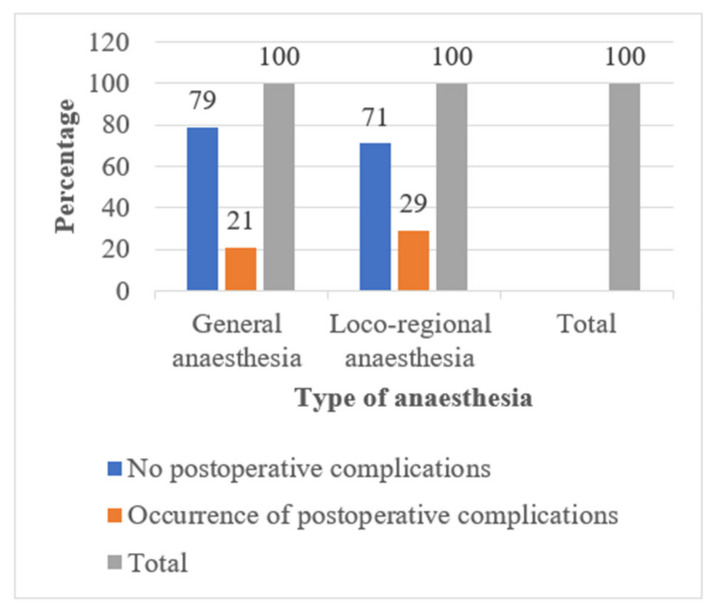
Incidence of post-operative complications according to the type of anaesthesia.

Opinions still vary as to the choice of anaesthetic technique for a parturient with sickle cell disease. Camous et al., in a study to determine the impact of anaesthetic technique on the occurrence of postnatal complications related to sickle cell disease, suggested that general anaesthesia may be associated with post-natal sickle cell complications, even when the severity of the disease has been taken into account [22]. Bakri et al., on the other hand, suggested that spinal anaesthesia may have advantages over general anaesthesia in parturients with sickle cell disease undergoing caesarean section [23]. Loco-regional anaesthesia is of particular interest and could be recommended for caesarean anaesthesia because providing a sympathetic block leads to vasodilation and increased peripheral microcirculatory flow rates and, thus, probably prevents vaso-occlusive events. Despite this apparent advantage of loco-regional anaesthesia, in our study, we did not observe a statistically significant difference between this anaesthesia technique and general anesthesia in terms of the occurrence of post-operative complications, regardless of the type of surgery, although the patients who received locoregional anaesthesia had more post-operative complications than those who received general anaesthesia (Figure 2).

The type of surgery could also be associated with the risk of post-operative complications. Griffin et al. conducted a retrospective study of 54 children undergoing 66 elective surgical procedures without pre-operative blood transfusion, and 10 children undergoing 10 elective procedures with pre-operative blood transfusion, over 16 years [24]. They found that patients who underwent laparotomy, thoracotomy, or tonsillectomy and adenoidectomy had a higher risk of developing post-operative complications. Contrary to this study, in our series, the type of surgery after which there were the most post-operative complications was orthopaedic surgery. There was no significant difference in impact between the type of surgery and the occurrence of post-operative complications.

The perioperative risk for patients with sickle cell disease is thought to be greatly increased in an emergency setting. The urgent nature of the surgery associated with other factors, such as hypoxia, hypothermia, dehydration, acidosis, and pain, increases the incidence of sickling in red blood cells [13]. In a large cohort of paediatric patients (813) with sickle cell disease undergoing abdominal surgery, urgent surgical procedures had almost twice the risk of complications as elective procedures [20]. These results contrast with ours, in which the context of surgical urgency was not predictive of post-operative complications, although the frequency of urgent surgeries was almost similar, with 13.7% in ours and 13% in theirs.

## 7. Elements That Had a Significant Impact on the Occurrence of Post-Operative Complications

Once the diagnosis of sickle cell disease is made at any time, it is important to organize the modalities of follow-up and management [25], which are focused on hydration, vaccination, pneumococcal antibiotic prophylaxis with penicillin V, systematic supplementation with folic acid, and hydroxycarbamide in case of repeated complications [26,27]. This follow-up could affect the patient’s condition before surgery and most likely the occurrence of post-operative complications. A study conducted between January 2009 and March 2012 on patients with sickle cell disease who had undergone cholecystectomy [18] showed that among the factors studied, a history of acute chest syndrome was associated with the occurrence of post-operative complications. In our study, the number of vaso-occlusive crises experienced per year was a significant predictor of the occurrence of post-operative complications. On one hand, this may imply that reducing the frequency of vaso-occlusive crises in patients with sickle cell disease should reduce the occurrence of post-operative complications but, on the other hand, this is also a warning that these patients should be more closely monitored post-operatively.

**Figure 3:**
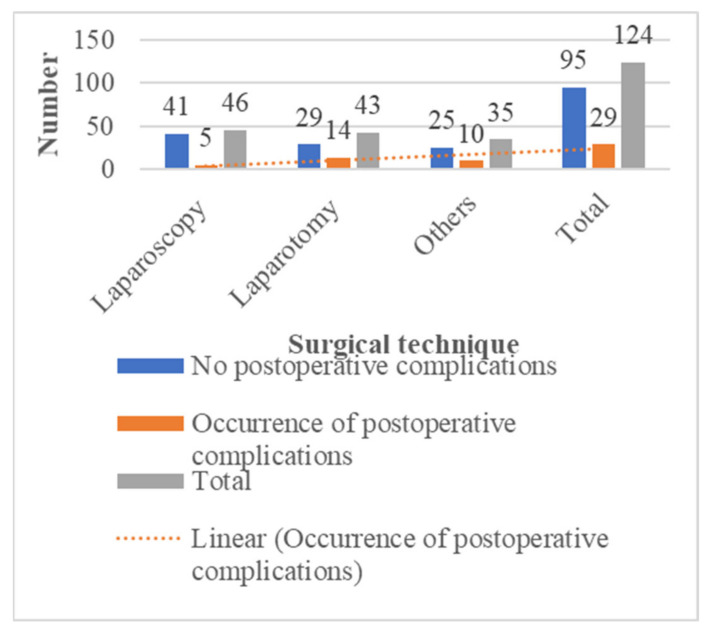
Incidence of post-operative complications according to surgical technique.

The surgical technique in our series was associated with post-operative complications, especially for abdominal surgical procedures. Patients who underwent laparoscopic surgery had fewer post-operative complications than those who underwent laparotomy (Figure 3). Laparoscopic surgery, particularly cholecystectomy, is considered a standard operative procedure, especially for gallstone disease (which is common in sickle cell disease). However, although many studies have been published on the benefits and complications of laparoscopic cholecystectomy in lithiasis cholecystitis [9,28,29,30], the evidence for the safety and efficacy of laparoscopy in the management of cholelithiasis in paediatric patients with sickle cell disease remains limited, and related controversies are unresolved. Moreover, laparoscopic surgery is not yet fully available in sub-Saharan African countries and training programs should be more intensively developed, which is now the case in Douala, Cameroon.

Our study had limitations. The types of surgery were varied in different proportions, and with different levels of risk. This could constitute a bias in the expression of certain characteristics of the less-represented procedures.

## 8. Conclusions

Sickle cell disease causes life-threatening complications in the peri-operative period and is of great interest to the anaesthetist. Although practices in the routine follow-up and perioperative management of sickle cell patients in resource-limited settings differ from those in high-resource settings, the incidence of post-operative complications and the complications encountered are not significantly different. Particular attention should be paid to female patients with sickle cell disease as they are more likely to experience post-operative complications, as well as to the frequency of vaso-occlusive crises, which are also predictive of post-operative complications. Pre-operative blood transfusion alone is not sufficient to reduce the occurrence of post-operative complications. Better management of sickle cell disease outside the acute phases, combined with the choice of laparoscopy as far as possible during abdominal surgical procedures, would make it possible to reduce the occurrence of post-operative complications.

## Figures and Tables

**Table 1:** Characteristics of the study population.

Total Number of Patients = 124	Number (*n*)	Percentage (%)
Genotype		
SS	112	90
SC	12	10
Usual follow-up		
Follow-up by a haematologist	24	19.3
Follow-up by another physician	44	35.5
On Hydroxyurea	26	21
Pre-operative transfusion	100	80.6
ASA classification		
ASA2	101	81.5
ASA3	23	18.5
Type of anaesthesia		
General anaesthesia	83	67
Loco-regional anaesthesia	41	33
Level of risk associated with surgery		
Intermediate	119	96
High	5	4
Surgical context		
Emergency surgery	17	13.7
Elective surgery	107	86.3
Type of surgery		
Digestive surgery	73	59
Orthopedic surgery	30	24
Obstetrical surgery	16	13
Otolaryngology surgery	5	4
Surgical technique		
laparotomy	43	34.7
Laparoscopy	46	37.1
Others	35	28.2
Post-operative complications recorded		
Fever	16	13
Vaso-occlusive crisis	15	12.1
Acute chest syndrome	3	2.4
Deglobalization with severe anaemia	5	4
Parietal suppuration	5	4
Eclampsia of post-partum	2	1.6

**Table 2:** Logistic regression dependence between the variables.

Variables	Coefficient	E.S	Wald	ddl	Sig.	Exp(B)
Sex	−1.431 **	0.579	6.104	1	0.013	0.239
On Hydroxyurea	−0.358	0.700	0.262	1	0.609	0.699
Number of VOC per year	0.499 **	0.239	4.373	1	0.037	1.647
ASA classification	0.338	0.792	0.182	1	0.670	1.402
Emergency context	0.291	0.813	0.128	1	0.721	1.338
Length of pre-operative hospital stay	−22.550	40,192.717	0.000	1	1.000	0.000
Pre-operative transfusion	−0.009	0.666	0.000	1	0.989	0.991
Type of surgery	1.873	1.454	1.659	1	0.198	6.507
Surgical technique	1.664 **	0.717	5.391	1	0.020	5.280
Anesthesia technique	−0.819	0.675	1.471	1	0.225	0.441
Duration of surgery	0.027	0.021	1.684	1	0.194	1.028
*Constant*	17.269	40,192.717	0.000	1	1.000	31,620,578.337
*Classification Table*	*81.5%*					
*Log* *likelihood*	*108.697*					
*Cox and Snell R-square*	*0.190*					
*Nagelkerke R-square*	*0.287*					
Khi-Square	26.194				0.051	

** are significant at 5%.
